# Comprehensive sequencing of the myocilin gene in a selected cohort of severe primary open-angle glaucoma patients

**DOI:** 10.1038/s41598-019-38760-y

**Published:** 2019-02-28

**Authors:** Luke O’Gorman, Angela J. Cree, Daniel Ward, Helen L. Griffiths, Roshan Sood, Alastair K. Denniston, Jay E. Self, Sarah Ennis, Andrew J. Lotery, Jane Gibson

**Affiliations:** 1 Human Development and Health, Faculty of Medicine, University of Southampton, Southampton, UK; 2 Clinical and Experimental Sciences, Faculty of Medicine, University of Southampton, Southampton, UK; 3 Molecular Genetics Wessex Regional Genetics Laboratory, Salisbury NHS Foundation Trust, Salisbury, UK; 4 Biological Sciences, Faculty of Natural and Environmental Sciences, University of Southampton, Southampton, UK; 5 Department of Ophthalmology, University Hospitals Birmingham NHS Foundation Trust, Birmingham, UK; 6 Eye Unit, University Hospital Southampton, Southampton, UK; 7 Human Genetics & Genomic Medicine, Faculty of Medicine, University of Southampton, Southampton, UK

## Abstract

Primary open-angle glaucoma (POAG) is the most common form of glaucoma, prevalent in approximately 1–2% of Caucasians in the UK over the age of 40. It is characterised by an open anterior chamber angle, raised intraocular pressure (IOP) and optic nerve damage leading to loss of sight. The myocilin gene (*MYOC*) is the most common glaucoma-causing gene, accounting for ~2% of British POAG cases. 358 patients were selected for next generation sequencing (NGS) with the following selection criteria: Caucasian ethnicity, intraocular pressure (IOP) 21–40 mm Hg, cup:disc ratio ≥0.6 and visual field mean deviation ≤−3. The entire *MYOC* gene (17,321 bp) was captured including the promoter, introns, UTRs and coding exons. We identify 12 exonic variants (one stop-gain, five missense and six synonymous variants), two promoter variants, 133 intronic variants, two 3′ UTR variants and 23 intergenic variants. Four known or predicted pathogenic exonic variants (p.R126W, p.K216K, p.Q368* and p.T419A) were identified across 11 patients, which accounts for 3.07% of this POAG cohort. This is the first time that the entire region of *MYOC* has been sequenced and variants reported for a cohort of POAG patients.

## Introduction

## Primary open-angle glaucoma

Glaucoma accounts for 7.9% of blindness in the UK^1^. It is characterised by a progressive loss of retinal ganglion cells, atrophy of the optic nerve and degradation of the visual field^2^. Glaucoma presents in multiple forms with primary open-angle (POAG) being the most common form^3,4^.

POAG is characterised by an open anterior chamber angle and raised intraocular pressure (IOP) leading to damage of the optic nerve and visual field loss^3^. POAG affects at least 1% of Caucasians in the UK over the age of 40 years^5^. Normal tension glaucoma (NTG) is a form of POAG in which optic nerve damage and visual field degradation are characteristic traits, however, IOP is not elevated^4^.

Approximately 5% of POAG is accounted for by monogenic, Mendelian-like variants. The myocilin gene (*MYOC*) accounts for the majority, approximately 2.2% of cases^6^. The optineurin gene (*OPTN*) may contribute to POAG in some populations, but has been implicated in normal tension glaucoma where is accounts for 1.5% of cases^7^. The majority of POAG cases are assumed to be accounted for by combined effects of multiple genetic and non-genetic risk factors. IOP is considered the most important risk factor in POAG. Other important risk factors in POAG include age, race, refractive error, central corneal thickness and family history of POAG^5,8,9^. However, these risk factors alone do not cause glaucoma^7^.

## Myocilin

*MYOC* is understood to have a role in cytoskeletal development and regulation of intraocular pressure (IOP)^10^. It is also known as the trabecular meshwork glucocorticoid-inducible response protein (TIGR)^10,11^. Variants in *MYOC* have also previously been identified as the cause of hereditary juvenile-onset open-angle glaucoma (JOAG), which is an early onset (<40 years) sub-set of POAG^12–15^.

*MYOC* is expressed in multiple tissues within the eye including the trabecular meshwork and ciliary body suggesting that it causes an increase in IOP through obstruction of the aqueous outflow^14,16^. It is also expressed at similar levels in a range of organs and tissue including the heart, skeletal muscle and bone marrow amongst others^17^.

The *MYOC* gene is encoded on the negative strand and comprises three exons. *MYOC* has one known RefSeq transcript (NM_000261.1) and spans 17,321 bp^18,19^. *MYOC* encodes a 504 amino acid polypeptide which consists of an N-terminal helix-turn-helix domain and two coil-coils^20^ and can homodimerise through leucine zipper interactions^21^. The C-terminal olfactomedin-like domain^22^ is part of a family of mucus proteins which are mainly found in nasal mucus^23^.

Known variants in *MYOC* are curated and made available online via the ‘myocilin allele-specific glaucoma phenotype database’^24^. Within this database, exons 1, 2 and 3 have 32, 1 and 62 known glaucoma-causing variants respectively. There are no glaucoma-causing variants annotated in the promoter, intronic or intergenic regions currently (27/11/2017)^24^. The database reports disease-causing variants comprising missense (83.7%), nonsense (5.8%), <21 bp deletion (4.8%), <21 bp insertion (4.8%) and <21 bp indels (1%). The Exome Aggregation Consortium (ExAC)^25^ scores the probability of loss-of-function intolerance (pLI) as 0.00 indicating that *MYOC* is tolerant of loss-of-function, and *MYOC* homozygous knockout mice experiments have excluded haploinsufficiency as a disease mechanism underlying POAG^26^. Shepard *et al*. suggested a gain-of-function is the likely cause of POAG and concluded p.Y437H variants in human *MYOC* induce exposure of an N-terminal cryptic peroxisomal targeting signal sequence^16^. The majority of pathogenic *MYOC* variants are found in exon 3^6,27^ where the most prevalent pathogenic variants are found to have a penetrance of up to 90%^17^. The concentration of known variants in exon 3 may have been exacerbated in recent years due to preferential analysis of this exon. Since the *MYOC* gene involves an autosomal dominant mode of inheritance in POAG^14^, pathogenic heterozygous variants would be a sufficient genotype for causality.

## Aim

In this study, the entire region of *MYOC* is assessed in 358 individuals with POAG selected from a UK cohort. Through the use of next-generation sequencing (NGS), application of bioinformatic tools and strategic filtering of variants, variants across the intergenic, promoter, UTR, exonic coding sequences and intronic regions are reported for the first time.

## Methods

Patients with Primary Open Angle Glaucoma (POAG) were recruited from eye clinics at University Hospital Southampton, Addenbrook’s Hospital Cambridge, Frimley Park Hospital Surrey, Queen Elizabeth Hospital Birmingham, Queen Alexandra Hospital Portsmouth, Romsey Hospital, St Mary’s Hospital Isle of Wight, Torbay Hospital Devon and New Cross Hospital Wolverhampton. Patient data was collected including gender, ethnicity, family history of POAG, specific diagnosis of the patient, age at diagnosis, intraocular pressure (IOP), cup:disc ratio (CDR), central corneal thickness and visual field mean deviation (VFMD). Blood samples were collected and DNA was extracted using the salting out method^28^ and stored at −20 °C. Initially, 372 patients were selected for Next Generation Sequencing (NGS) using the following selection criteria: Caucasian ethnicity, 21 mm Hg ≤ IOP ≤ 40 mm Hg, cup:disc ratio ≥0.6 and visual field mean deviation ≤−3. Ten patients who passed all inclusion criteria also had one affected first degree relative recruited to the study, even if they did not meet all inclusion criteria.

The entire *MYOC* gene was targeted for inclusion using a custom sequencing panel to include the intronic, exonic, UTR and promoter regions (Table 1) and an additional 1000 bp upstream of the Eukaryotic Promoter Database defined promoter coordinates (hg38 coordinates: chr1:171652678–171652737)^29,30^.

**Table 1:** *MYOC* promoter, intronic, exonic and intergenic region locations within hg38 human reference genome.

	Chromosome	Start	End	Length (bp)
Promoter	1	171652678	171652737	59
Prom-5′ UTR	1	171652633	171652678	45
5′ UTR	1	171652611	171652633	22
Exon 1	1	171652007	171652611	604
Intron 1	1	171638722	171652007	13285
Exon 2	1	171638596	171638722	126
Intron 2	1	171636709	171638596	1887
Exon 3	1	171635924	171636709	785
3′ UTR	1	171635416	171635924	508

Library preparation and sequencing were performed in local laboratories, where DNA was simultaneously fragmented and tagged with sequencing adapters using the Illumina’s Nextera Rapid Capture Custom Enrichment kit (Illumina 5200 Illumina Way San Diego, California USA). Target regions of DNA were bound and amplified with custom capture probes and enriched prior to running on a Illumina NextSeq500 sequencing machine. Sequencing was performed in three batches of 96 samples and one batch of 84 samples.

Next generation sequencing (NGS) data were aligned against the human reference genome (hg38) using BWA-mem^31^. Variant calling was performed using GATK v3.7^32^. Annotation was performed with ANNOVAR^33^ against a database of RefSeq transcripts^19^, Exome Aggregation Consortium (ExAC)^25^, 1000 Genomes Project^34,35^ and conservation-based pathogenicity scores of sort intolerant from tolerant (SIFT)^36^, PhyloP, PhastCons^37^ and Genomic Evolutionary Rate Profiling (GERP++)^38^. Variants were also annotated with MutPred Splice^39^ and Human Splicing Finder v3.0 (HSF3.0)^40,41^ to evaluate disruption of splicing. Further annotation was performed using non-coding Functional Analysis through Hidden Markov Models (FATHMM)^42,43^ and Combined Annotation Dependent Depletion (CADD)^44^, and incorporation of the ‘Myocilin allele-specific glaucoma phenotype database’^24^.

For the 372 sequenced patient samples, coverage across both the *MYOC* gene and all targets were determined. The proportion of variants shared between samples was checked for consistency with known sample relationships and ethnicities. VerifyBamID v1.1.13^45^ software was used to estimate possible contamination and a ‘freemix’ value threshold of >0.03 was applied^46^. Coverage statistics were generated using SAMtools v1.3.1^47^ and BEDtools v2.17.0^48^. A minimum threshold of 20X depth was used to distinguish samples with sufficient coverage.

For the entire region of the *MYOC* gene, depth per base was calculated with SAMtools v1.3.1^47^ and conservation scores were downloaded from the University of California Santa Cruz (UCSC) database for PhyloP^37^ and PhastCons^37^ databases of 20 mammals. Regions were considered in ‘high conservation’ if PhastCons ≥0.4^49^ or PhyloP ≥1.5^50^. Repetitive regions were annotated using UCSC RepeatMasker data, and rare variants (allele frequency ≤ 0.05) in the POAG cohort and 1000 Genomes Project were plotted.

Variants were considered as previously known glaucoma-causing variants if they were identified as ‘Glaucoma-causing’ in the ‘Myocilin allele-specific glaucoma phenotype database’ or ClinVar. Exonic variants were prioritised as candidate causal variants through CADD Phred scores ≥15^51^. Exonic splice variants were prioritised if they exceeded a 0.6 MutPred Splice score threshold^39^.

Non-coding variants were prioritised using FATHMM which out-performs CADD in the non-coding region whilst CADD has a superior classifier over FATHMM in the coding region. Variants were prioritised if the FATHMM score exceeded the default threshold of ≥0.5^43^. Intronic variants were also prioritised if they were flagged as potentially splice affecting in HSF3.0^40,41^.

CNVkit, which is designed for use with custom target panels and short-read Illumina sequencing, was used to infer copy number^52^. Data were analysed in batches to account for variation in average depth between batches, and a pooled reference of all samples within the batch was used.

Consent was obtained in accordance with the Declaration of Helsinki and was approved by South West Hampshire Local Research Ethics Committee (05/Q1702/8). Informed consent was obtained from all subjects, and all methods were carried out in accordance with the relevant guidelines and regulations of Research Ethics Committees (REC).

## Results

### Samples analysed

358 of 372 patients passed inclusion criteria after sample quality control. One sample was omitted due to insufficient depth (≤20X), five were omitted due to post hoc detection of sample duplication or mixed race individuals. A further eight samples were omitted due to the patient age at diagnosis being less than 40 years. Selected demographic and clinical characteristics for all 358 individuals in the final analysis are summarised in Table 2.

**Table 2:** Demographic and clinical characteristic summaries of age, intraocular pressure (IOP), cup:disc ratio (CDR) and visual field mean deviation (VFMD) for POAG cohort (n = 358).

	Min	Max	Mean	Median	SD
Age (years)	42	91	66	66	11
IOP (mmHg)	21.00	42.00	27.90	27.00	4.67
CDR	0.60	1.00	0.82	0.80	0.09
VFMD	−31.54	−1.07	−14.52	−14.51	7.68

**Figure 1:**
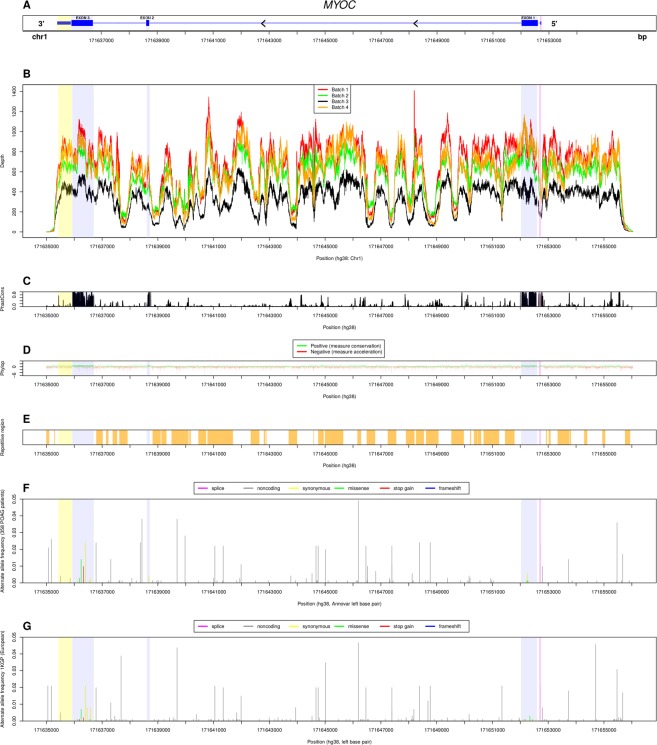
Per base analysis of variants and regions across the *MYOC* gene. Per base analysis of read depth, conservation scores, repetitive region context and allele frequency across the POAG patient data set (n = 358) for *MYOC*. (**A**) Gene structure of *MYOC*. (**B**) Per base depth for samples of batch 1, red; batch 2, green; batch 3, black; batch 4 orange;. (**C**) PhastCons 20 way mammals conservation score (20 mammals). (**D**) Phylop scores (20 mammals), showing measure of conservation (green) and acceleration (red). (**E**) Regions identified as repetitive regions by RepeatMasker (orange). (**F**) Allele frequency for each base’s detected alternate allele across 358 POAG samples (left base position). (**G**) Allele frequency of SNPs in 1000 Genomes Project (European) for corresponding alternate alleles identified in F.

### Genomic features and variation across the *MYOC* gene

The coordinates of the *MYOC* gene (5′ promoter - 3′ UTR) are chr1:171,652,737–171,635,416 (hg38). Coverage was uninterrupted across the entire region with 100% coverage at 20X depth for all samples. The four batches had mean depths of 717X, 552X, 389X, and 726X across target regions respectively, averaged across all samples. The poorest coverage was observed in batch 3. A consistent coverage pattern is seen for all four batches (Fig. 1B) and was found to be correlated with mappability, repetitive context, conservation and GC content (R^2^ = 0.3358, p-value < 2.2 × 10^−16^). Conservation scores derived from PhastCons (Fig. 1C) and PhyloP (Fig. 1D) were highest across exonic regions, with smaller regions of high conservation in intron 1 and a region upstream of the Eukaryotic Promoter Database defined promoter region.

We identified a total of 172 annotated variants comprising 160 SNPs and 12 indels in the POAG cohort of 358 individuals (Table 3). These variants were distributed across *MYOC* with 21 variants upstream intergenic, two variants in the promoter region, four in exon 1, 118 in intron 1, one in exon 2, 15 in intron 2, seven in exon 3, two in the 3′ UTR and two variants in the downstream intergenic region. 156 SNPs were identified in the non-coding regions of the *MYOC* gene in the POAG cohort. The majority (70.5%) of non-coding variants were located in the largest intron, intron 1, which spans 13,285 bp (76.7%) of the 17,321 bp length of *MYOC*. For comparison, there were 574 total SNPs in the 1000 Genomes Project European population (1000gEUR) in *MYOC*. There were 105 rare (AF < 5%) variants in POAG and 134 rare (AF < 5%) variants in 1000gEUR, and these had a similar distribution across the *MYOC* gene (Fig. 1F and G).

**Table 3:** Summary of the number of variants identified across all features of the *MYOC* gene.

Gene feature	No. variants		
	No. unique variants	SNPs	Indels
Intergenic us	21	21	0
Promoter	2	2	0
Promoter-5′ UTR	0	0	0
5′ UTR	0	0	0
Exon 1	4	4	0
Intron 1	118	110	8
Exon 2	1	1	0
Intron 2	15	12	3
Exon 3	7	7	0
3′ UTR	2	2	0
Intergenic ds	2	1	1
All	172	160	12

Three SNPs were identified with high conservation in PhastCons (20 mammals), using a threshold of 0.4, and excluding variants in repetitive regions. The variant rs76745622 was located in the upstream intergenic region whilst rs11586716 and rs12035960 were located in intron 1. No variants were identified in the non-coding regions with high conservation using PhyloP (≥1.5).

### Exonic variants

A total of 12 exonic variants were called (Table 4). Four SNPs were detected in exon 1, one SNP in exon 2 and seven SNPs in exon 3. Four variants had CADD Phred scores greater than 15 suggesting the variants were likely pathogenic. Variants NM_000261:exon3:c.C1102T (p.Q368*) and NM_000261:exon1:c.C376T (p.R126W) had previously been identified as ‘Glaucoma-causing’ by the ‘myocilin allele-specific glaucoma phenotype database’. Variant p.R126W also had a high MutPred splice score of 0.605, indicating that this variant was likely to affect splicing.

**Table 4:** Annotation of all exonic variants in *MYOC*.

No.	Exon	Chrom	POS	Ref	Alt	Variant type	Amino acid	dbSNP144	1000 G EUR	ExAC NFE	Sample count	Study AF	myocDB	CLINSIG	SIFT	gerp++ gt2	PhastCons	CADD Phred	MutPred Splice
1	1	chr1	171,652,385	C	T	nonsynonymous	R76K	rs2234926	0.14120	0.13650	92	0.13700	Neutral	Benign	0.049	3.43	0.945	9.00	0.116
2	1	chr1	171,652,269	C	T	nonsynonymous	E115K	rs757551979	—	0.00003	1	0.00140	—	—	0.589	3.53	0.268	9.37	0.159
3	1	chr1	171,652,246	G	A	synonymous	G122G	rs145354114	—	0.00300	4	0.00559	Neutral	Uncertain	—	—	0.000	0.17	0.494
**4**	**1**	**chr1**	**171**,**652**,**236**	**G**	**A**	**nonsynonymous**	**R126W**	**rs200120115**	—	**0**.**00007**	**1**	**0**.**00140**	**Glaucoma**	—	**0**.**019**	−**1**.**13**	**0**.**008**	**23**.**50**	**0**.**605**
**5**	**2**	**chr1**	**171**,**638**,**679**	**C**	**T**	**synonymous**	**K216K**	**rs141584495**	—	**0**.**00050**	**3**	**0**.**00419**	**Neutral**	—	—	—	**0**.**992**	**15**.**63**	**0**.**165**
6	3	chr1	171,636,585	C	A	synonymous	T285T	rs146606638	0.00800	0.00480	2	0.00279	Neutral	Benign	—	—	0.591	14.00	0.126
7	3	chr1	171,636,534	G	A	synonymous	D302D	rs148433908	—	0.00030	1	0.00140	Neutral	—	—	—	0.000	0.07	0.137
8	3	chr1	171,636,399	A	G	synonymous	Y347Y	rs61730974	0.02090	0.03050	17	0.02400	Neutral	—	—	—	0.024	0.00	0.140
**9**	**3**	**chr1**	**171**,**636**,**338**	**G**	**A**	**stopgain**	Q368*	**rs74315329**	**0**.**00200**	**0**.**00150**	**7**	**0**.**00978**	**Glaucoma**	**Pathogenic**	—	**4**.**52**	**0**.**283**	**37**.**00**	**0**.**374**
10	3	chr1	171,636,247	T	C	nonsynonymous	K398R	rs56314834	0.00700	0.00480	10	0.01400	Neutral	—	0.618	−1.17	0.843	3.87	0.268
**11**	**3**	**chr1**	**171**,**636**,**185**	**T**	**C**	**nonsynonymous**	**T419A**	—	—	—	**2**	**0**.**00279**	—	—	**0**.**000**	**5**.**04**	**0**.**945**	**23**.**50**	**0**.**196**
12	3	chr1	171,636,126	G	A	synonymous	T438T	rs375235405	—	0.00004	1	0.00140	—	—	—	—	0.898	11.66	0.125

Exon, exon number; Feature, genetic feature within *MYOC*; Chrom, chromosome; POS, location of 5′ base of variant in hg38; Ref, reference allele; Alt, alternate allele; Variant type, type of variant observed; Amino Acid, amino acid single letter abbreviation of reference amino acid and the amino acid substituted to; dbSNP144, rs ID if the variant is known; 1000 G EUR, allele frequency from 1000 Genomes Project (European ethnic sub-group); ExAC NFE, allele frequency from ExAC Non-Finnish European ethnic sub-group; Sample count, number of patients with the variant in the n = 358 POAG cohort; Study AF, allele frequency of the variant within the n = 358 POAG cohort; myocDB, known *MYOC* variants database^24^; CLINSIG, pathogenicity of the variant in ClinVar; SIFT, sorts intolerant from tolerant substitutions; gerp++, Genomic Evolutionary Rate Profiling; PhastCons, conservation scoring and identification of conserved elements; CADD Phred, Combined Annotation Dependent Depletion on a Phred scale; MutPred Splice, machine learning-based predictor of exonic splice variants. Bold indicates variants which are causal candidates.

Two variants NM_000261:exon2:c.G648A (p.K216K) and NM_000261:exon3:c.A1255G (p.T419A) not previously identified as glaucoma-causing, had pathogenicity scores indicating they may be of importance. In three individuals p.K216K had a high PhastCons score of 0.992 indicating that it is within a highly conserved element. The variant was also more common in the POAG cohort (AF = 0.0042) than in the ExAC Non-Finnish European (NFE) population (AF = 0.0005).

The missense variant p.T419A had no known rsID and was not found in ExAC NFE. In the POAG cohort it has an allele frequency of 0.0028, and identified as heterozygous in two individuals. It is located in exon 3 and has a SIFT score of 0, PolyPhen HDIV score of 0, GERP++ score of 4.52 and CADD Phred of 37 which indicate that this variant is likely to be highly pathogenic. However, this variant was found to be present on the same read pair as the p.Q368* variant in both patients (see Supplementary Fig. S1).

There was no significant difference in sub-phenotypes between patients with candidate causal *MYOC* variants (p.Q368*, p.R126W, p.K216K or p.T419A) and patients with no candidate causal *MYOC* variants (t-test, IOP p-value = 0.766, CDR p-value = 0.626, VFMD p-value = 0.211). However, hypertension was treated in five of the 11 patients with candidate causal *MYOC* variants.

**Figure 2:**
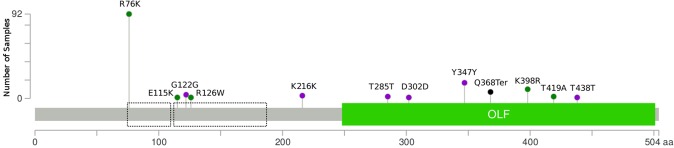
Human MYOC protein structure with its domains and variants mapped^67,68^. Additional annotation of coiled-coil domains are outlined in black (aa74-110 and aa118-186^20^). Green dots indicate a missense variant, purple dots indicate synonymous variants, whilst black dots indicate stop-gains.

Three variants, p.E115K, p.G122G and p.R126W are clustered within the coiled-coil located at aa118-aa186^20^ (Fig. 2). The aa117-aa166 region contains lysine residues responsible for dimerisation of *MYOC*^53^. The p.K216K variant is located in a linker region whilst p.T285T, p.D302D, p.Y347Y, p.Q368*, p.K398R, p.T419A and p.T438T are all located within the large olfactomendin-like domain.

### Non-coding variants

There were 160 variants identified in non-coding regions (see Table 3 and Supplementary Fig. S2). The majority of variants (118) were identified within the largest region, intron 1.Using a FATHMM threshold of 0.5 to prioritise the non-coding variants, one variant upstream of the promoter, three intron 1 and one intron 2 variants remain (Table 5). The highest FATHMM score of 0.86 was seen for a common variant with an allele frequency of 10% in the 1000gEUR, and similar (8.2%) in the POAG cohort. A single variant in intron 1, NM_000261.1:c.605-5949C>T, had a CADD Phred score greater than 15, however, there was no significant difference in frequency between this variant in the POAG cohort compared with the 1000gEUR cohort (allelic chi-squared test, p-value = 0.716). A second intron 1 variant, NM_000261.1:c.604+5942G>A, had a CADD Phred score of 12.78, a GERP++ score of 3.57 and PhastCons score of 0.504. Although pathogenicity scores are in favour of a potentially pathogenic effect, allele frequencies show no significant difference between the POAG cohort and 1000gEUR (allelic chi-squared test, p-value = 0.132). The novel intergenic upstream variant identified, NM_000261.1:c.-2851C>T, had a CADD Phred score of 11.19, a GERP++ score of 3.22 and low conservation PhastCons score of 0.0079 providing ambiguous indications of pathogenicity. This variant is not present in the 1000gEUR but is observed as heterozygous in one individual within the POAG cohort.

**Table 5:** Five non-coding variants in the *MYOC* region remain following initial filtering of the 160 non-coding variants with FATHMM ≥0.5.

No.	Feature	Chrom	POS	Ref	Alt	dbSNP144	1000 G EUR	Sample count	Study AF	gerp++ gt2	FATHMM	CADD Phred	myocDB	Repeat	PhastCons	Regulatory build
1	INTERGENIC_US	chr1	171655462	C	T	—	—	1	0.00140	3.22	0.593	11.19	—	0	0.008	CTCF Binding Site
2	INTRON1	chr1	171,646,066	C	T	rs12035960	0.10440	55	0.08200	3.57	0.860	12.78	—	0	0.504	Promoter Flanking Region
3	INTRON1	chr1	171,644,671	G	A	rs75953590	0.01990	16	0.02200	—	0.551	15.38	—	0	0.244	—
4	INTRON1	chr1	171,643,942	G	C	rs144750384	0.00800	1	0.00150	—	0.623	10.31	—	1	0.061	Open chromatin
5	INTRON2	chr1	171,637,310	A	G	rs79263003	0.01090	10	0.01400	—	0.570	4.669	—	0	0.280	—

Feature, genetic feature within *MYOC*; Chrom, chromosome; POS, location of 5′ base of variant in hg38; Ref, reference allele; Alt, alternate allele; dbSNP144, rsID if the variant is known; 1000 G EUR, allele frequency from 1000 Genomes Project (European ethnic sub-group); Sample count, number of patients with the variant in the n = 358 POAG cohort; Study AF, allele frequency of the variant within the n = 358 POAG cohort; gerp++, Genomic Evolutionary Rate Profiling; FATHMM, Functional Analysis through Hidden Markov Models; CADD Phred, Combined Annotation Dependent Depletion on a Phred scale; myocDB, known *MYOC* variants database^24^; Repeat, repetitive region as defined by RepeatMasker; PhastCons, conservation scoring and identification of conserved elements; Regulatory build, Ensembl Regulatory Build containing regions that are likely to be involved in gene regulation.

20 variants were flagged as ‘potentially splice altering’ by human splice finder (HSF) version 3.0, 18 of which had rsIDs (see Supplementary Table S2). 17 splice variants were in intron 1 (15 SNPs, and 2 insertions) and three in intron 2 (three SNPs). Of these, three variants had potential to introduce both a new splice acceptor and/or a splice donor site (AD), nine introduced a new slice acceptor site only (A), seven a new donor site only (D) and one breaks a branch point (BBP).

No copy number variants (CNVs) were detected within the *MYOC* gene region. There were no losses in copy number (CN) across the entire region, however, some (N = 113) samples were called as a single copy gain with a CN = 3 across the entire region.

## Discussion

We have performed targeted next-generation sequencing on the full region of the *MYOC* gene (promoter, UTRs, coding exons, introns and intergenic regions) on 358 POAG patients with severe POAG sub-phenotypes. We report all variants detected across the region and have performed an in silico analysis to assess pathogenicity. We identified a known pathogenic stop-gain in exon 3, a known pathogenic missense variant in exon 1, a known synonymous variant not previously considered pathogenic in exon 2, and an unknown missense variant in exon 3. Between them these variants account for 11/358 (3.07%) of patients within our POAG cohort.

NM_000261:exon3:c.C1102T (p.Q368*) is the most common causal variant in POAG, accounting for 31.2% of disease-causing variants in *MYOC*^24^. This stop-gain is 6.5 times more common in our POAG cohort than in the 1000gEUR (allelic Fisher’s Exact test, p-value = 0.0336) and is seen in seven patients, accounting for 63.6% of the candidate causal variants identified. Of the seven patients with p.Q368* variants, three did not have a positive family history of POAG in our database. Craig *et al*. have previously shown a 100% family history for this variant, however this was only after retrospective follow up and new diagnoses were made. They found that the index patient was sometimes unaware of their family history of disease and thus family history reported may be underestimated, which may also be that case here. Furthermore, the penetrance of this variant is high but not complete. 82% of carriers of p.Q368* have POAG or OHT at the age of 65 years, and an unaffected carrier has been identified at 74 years of age^54^. Shepard *et al*. have previously shown that protein MYOC monomers with this variant do not contain a cryptic peroxisomal signalling sequence (PTS1) and that it likely exposes the PTS1 sequence in its dimer partner^16^. This is believed to cause the mutant dimer to associate with the PTS1R and ultimately cause deleterious trabecular meshwork cell function^16^. This mechanism has been supported by other studies^55,56^. Previous studies have shown that Caucasian patients with the p.Q368* variant have a mean IOP ranging between 27.3–35.4 mm Hg^6,57–59^, higher than in our study (mean IOP = 26.3 mm Hg). In our study, patients with this variant had a mean CDR of 0.84, a mean VFMD of −13.84, and a mean age of 69.6 years. These findings agree with Graul *et al*. who found that p.Q368* patients did not have an earlier onset nor did they have a higher IOP^57^.

The known glaucoma-causing variant, NM_000261:exon1:c.C376T (p.R126W) was found in one of the 358 POAG patients and had been previously reported as a late-onset familial variant^60^. It is a variant which is located on the protein dimer region of a coiled-coil. Gobeil *et al*. have shown cell adhesion properties were unaffected^55^ by this variant. NM_000261:exon1:c.C376T (p.R126W) had damaging SIFT and CADD Phred scores of 0.019 and 23.5 respectively. There was very little evidence for splicing variation leading to POAG and only one previously known instance in *MYOC* of a predicted cryptic splice site reported within intron 1^61^. However, a MutPred Splice score of 0.605 implicated that this variant contributes to the creation of a new donor splice site and a subsequent loss of 372 nucleotides from exon 1. This finding suggests that splice variants could be more important in POAG than previously known. Faucher *et al*. has previously shown that patients with this variant were found to have a mean IOP of 28.3 mm Hg and an age of onset of 74^60^. The patient with this variant in our cohort showed similar traits with a maximum IOP of 27 mm Hg and an age at diagnosis of 74 (see Supplementary Table S3).

The synonymous variant NM_000261:exon2:c.G648A (p.K216K) was not previously considered a pathogenic variant. This variant is found in exon 2 which contains just one known pathogenic variant and is believed to translate to a linker region within the MYOC protein^24,62^. Synonymous variants in *MYOC* have been suggested to have a role affecting *MYOC* mRNA structure and subsequently the translated protein stability^63^. Variant p.L215Q, on the preceding codon of p.K216, is believed to be glaucoma-causing on the basis of an in-silico damaging SIFT score^62^. Similarly, p.K216K has strong in silico pathogenicity scores to suggest possible pathogenic status (PhastCons of 0.992 and CADD Phred of 15.63). Furthermore, this variant is found in the gnomAD Non-Finnish European (NFE) population^25^ significantly less frequently than the POAG cohort (allelic Fisher’s Exact test, p-value = 0.0109). This heterozygous variant was found in three patients from the University Hospital Southampton site. No evidence of relatedness was identified, however, there is a possibility that there is some distant relatedness which we do not have the capability to detect.

The missense variant NM_000261:exon3:c.A1255G (p.T419A) does not have an associated rsID, nor is it found within 1000gEUR or ExAC. However, it is found at an allele frequency of 8.952e-6 in the gnomAD NFE population. This variant has never been observed in a glaucoma context before but is seen as a heterozygote in two patients in this study (AF = 0.0028). This is a substantially higher frequency than gnomAD NFE (allelic Fisher’s Exact test, p-value = 9.4e-6). This variant had a SIFT score of 0, GERP++ score of 5.04, PhastCons of 0.945 and CADD Phred score of 23.5 which indicated further support for pathogenicity. However, we have found that in both patients p.T419A is co-inherited with the upstream p.Q368* variant (see Supplementary Fig. S1), therefore the protein will be truncated before translation of the potentially pathogenic substitution. Although these two patients had the earliest onset (50 & 56 years) of those carrying the p.Q368* variant, it is not possible to provide a plausible mechanism by which this variant could have a modifying effect.

Whilst there were no clear likely pathogenic variants in the non-coding region of the gene, NM_000261.1:c.-2851C>T which is located in the upstream intergenic region (44694 bp from the neighbouring *VAMP4* gene) was found to be of potential interest. Whilst it is not within a conserved element in PhastCons, it had damaging GERP++ and FATHMM scores. It has an allele frequency in the POAG cohort of 0.0014 (one heterozygous patient). This variant is not found within the 1000gEUR and is at a position not currently covered by gnomAD. The Ensembl regulatory build indicates that this variant could be functionally important as it is located at a potential CTCF binding site. All other variants with a FATHMM score ≥0.5 were seen at similar frequencies in both the POAG cohort and 1000gEUR. Genotyping this non-coding variant across a wider POAG cohort could prove informative. Variants located up to 1000 bp upstream of *MYOC* have been implicated as potentially functionally important for controlling IOP^64,65^.

Five rare variants were present in six individuals that potentially affect splicing. However, the presence of these variants in the European population cannot be confirmed due to lack of coverage in allele frequency databases for these sites.

We found no evidence of sub-gene copy number changes, and no whole gene deletions. We did predict some whole gene single copy gains and we suspect the predicted gain reflects within-batch depth variation. Patient selection criteria for this study used strict sub-phenotype parameters in order to select most severe POAG sub-phenotypes with a greater chance of an accurate POAG diagnosis. However, such criteria hinders genotype-phenotype analyses within the selected cohort. Genotyping of a larger POAG cohort not selected on sub-phenotypes is necessary in order to perform robust genotype-phenotype analyses. The *MYOC* gene accounts for ~3% of patients with POAG, therefore a larger cohort would also have greater power to detect rarer causal variants.

## Conclusion

For the first time all regions of *MYOC* have been sequenced and analysed in a POAG cohort. We have identified two known pathogenic variants and two high pathogenic scoring variants, which may cause POAG in 11 patients. Synonymous and non-coding variants have been identified as having pathogenic qualities using in silico pathogenicity predictions, and a known glaucoma-causing variant has been implicated as a potential deep exonic splice variant. This work expands the known allelic diversity of *MYOC* in POAG which is useful for diagnosis, genetic counselling and cascade genetic testing in families. Additional sequencing of *MYOC* interacting partners^66^ and other POAG-causing genes could reveal rare causal variants and provide further insight into the genetic basis of POAG.

## Supplementary information

Fig S1, Table S1 and Table S3

S2 Table

## Data Availability

Data generated or analysed during this study are included in this published article and its supplementary files.
