# Supplementary material for: Comprehensive sequencing of the myocilin gene in a selected cohort of severe primary open-angle glaucoma patients
Source: Sci Rep. 2019 Feb 28;9:3100. doi: 10.1038/s41598-019-38760-y (PMC6395666; doi:10.1038/s41598-019-38760-y)
Supplement: Supplementary file 1 — Fig S1, Table S1 and Table S3 [file 41598_2019_38760_MOESM1_ESM.pdf]

Comprehensive sequencing of the myocilin gene in a selected cohort of severe primary open-angle glaucoma patients

Luke O'Gorman[1], Angela J Cree[2], Daniel Ward[3], Helen Griffiths[2], Roshan Sood[4], Alastair K Denniston[5], Jay Self[2,6], Sarah Ennis[7*], Andrew J Lotery[2,6+], and Jane Gibson[4+]

[1]Human Development and Health, Faculty of Medicine
[2]Clinical and Experimental Sciences, Faculty of Medicine
[3]Molecular Genetics Wessex Regional Genetics Laboratory, Salisbury NHS Foundation Trust
[4]Biological Sciences, Faculty of Natural and Environmental Sciences
[5]Department of Ophthalmology, University Hospitals Birmingham NHS Foundation Trust
[6]Eye Unit, University Hospital Southampton
[7]Human Genetics & Genomic Medicine, Faculty of Medicine, University of Southampton

[*]se@soton.ac.uk
[+]Joint last authorship

Supplementary Information

S1 Fig. ***The Integrative Genomics Viewer (IGV) image of two samples with two variants, Q368* and T419A.*** *Both variants are found on the same read pair.*

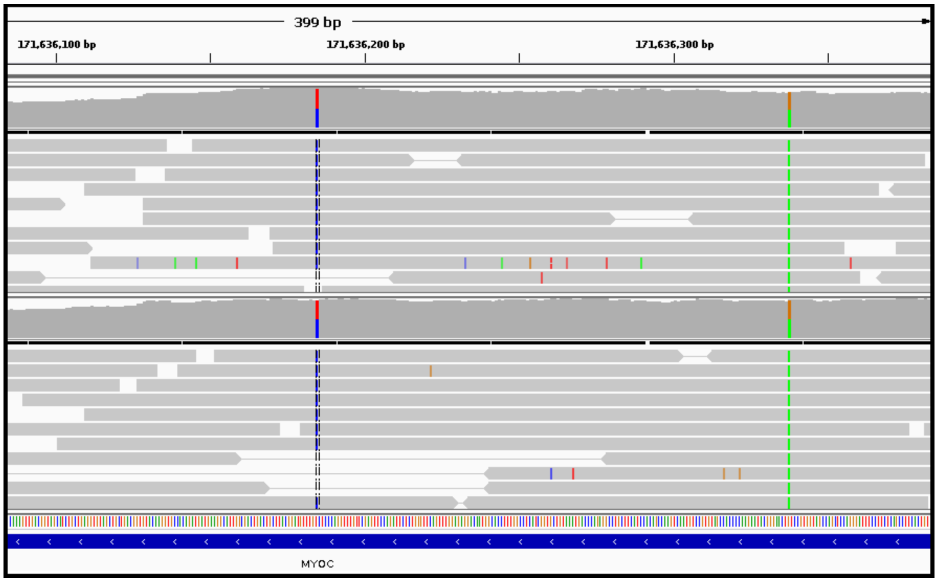

S2 Table. ***All variants identified in the MYOC gene within the study cohort (S2_Table.xlsx).***

S3 Table. ***11 cases carrying a candidate disease-causing MYOC mutation.*** *Demographic data and clinical data for each patient is listed including patient gender, ethnicity, age at diagnosis, family history of POAG, diagnosis, intraocular pressure (IOP), cup:disc ratio (CDR), central corneal thickness (CCT), visual field mean deviation (VFMD), cataract status and age-related macular degeneration (AMD) status.*

Patient	Exon	Variant	Study Site	Patient gender	Ethnicity	Age at diagnosis	Family history of POAG	Diagnosis	IOP	CDR	CCT	VFMD	Cataract	AMD
1	1	R126W	Frimley Park	Female	Caucasian	74	Yes	POAG	27	0.6	572	-4.8	Yes	No
2	2	K216K	University Hospital Southampton	Male	Caucasian	62	Yes	POAG	32	0.85	Not measured	-7.39	No	No
3	2	K216K	University Hospital Southampton	Male	Caucasian	65	Yes	POAG	34	0.7	569	-4.26	No	No
4	2	K216K	University Hospital Southampton	Female	Caucasian	68	Yes	POAG	23	0.9	588	-12.58	Yes	No
5	3	Q368*	Frimley Park	Female	Caucasian	87	No	POAG	24	0.9	529	-16.63	Yes	Yes
6	3	Q368*	Frimley Park	Female	Caucasian	85	No	POAG	23	0.8	534	-14.74	No	No
7	3	Q368*	University Hospital Southampton	Male	Caucasian	56	Yes	POAG	27	0.8	Not measured	-8.46	No	Yes
8	3	Q368*	Portsmouth	Male	Caucasian	74	No	POAG	24	0.8	487	-30.83	Yes	Yes
9	3	Q368*	Torbay	Female	Caucasian	79	Yes	POAG	30	0.95	Not measured	-13.58	Unknown	Unknown
10	3	Q368* & T419A	Birmingham	Female	Caucasian	50	Yes	POAG	30	0.8	Not measured	-3.34	Yes	No
11	3	Q368* & T419A	Portsmouth	Male	Caucasian	56	Yes	POAG	26	0.8	543	-9.3	Yes	No
